# Association between HPV vaccine-related social media discourse and regional HPV vaccine coverage in China

**DOI:** 10.1016/j.jve.2026.100639

**Published:** 2026-10-29

**Authors:** Zhongwen Wang, Xinsheng Wu, Jinyuan Wu, Guozhen Wu, Bin Li, Huachun Zou

**Affiliations:** a Shanghai Institute of Infectious Disease and Biosecurity, School of Public Health, Fudan University, 131 Dong'an Road, Shanghai, 200032, China; b Center for AIDS Research (CFAR), School of Public Health, Key Laboratory of Public Health Safety, Ministry of Education, Fudan University, Shanghai, China; c School of Public Health, Southwest Medical University, Luzhou, China; d Melbourne Sexual Health Centre, Faculty of Medicine Nursing and Health Sciences, Monash University, Melbourne, Australia

**Keywords:** Human papillomavirus (HPV), Vaccination, Social media, China

## Abstract

**Introduction:**

China's human papillomavirus (HPV) vaccination rate remains far below World Health Organization (WHO) targets. This study examined whether HPV vaccine discourse on Weibo is associated with regional vaccine coverage in China.

**Methods:**

We collected HPV vaccine–related Weibo posts from January 2022 to December 2022, with additional posts from January 2025 to December 2025 for temporal comparison. We fine-tuned a Chinese-RoBERTa-wwm-ext model to classify post themes, enabling us to identify discourse focuses and distinguish the nature of its association with coverage. Multivariate regression with nested leave-one-out cross-validation was performed to assess the association between Weibo post related variables and regional coverage rates, comparing against models using socioeconomic indicators (economy, education, healthcare, employment).

**Results:**

A total of 29,638 valid posts were included in the primary analysis. Approximately 30% of the posts were related to “vaccine promotion” and “vaccination experiences”, making them the two most frequent themes. Total post volume correlated strongly with HPV vaccine coverage (one-dose: *r* = 0.869, 95% confidence interval [0.744, 0.936], *P* < 0.001; three-dose: *r* = 0.920 [0.840, 0.961], *P* < 0.001), exceeding all included socioeconomic factors. Total post volume models outperformed socioeconomic models (LOOCV *R*^*2*^: one-dose 0.643 vs 0.606; three-dose 0.818 vs 0.662). After excluding retrospective themes, potentially prospective posts plus socioeconomic factors improved prediction (LOOCV *R*^*2*^: one-dose 0.620 vs 0.593; three-dose 0.797 vs 0.720). In 2025, posts decreased by 48.1% and decline in posts related to Theme 1 was the most pronounced.

**Conclusion:**

HPV vaccine discourse on Weibo was associated with regional coverage. Social-media signals deliver supplementary information beyond conventional socioeconomic indicators, which could serve as a complementary public-health surveillance tool to identify regional needs and barriers.

## 1 Introduction

Cervical cancer is one of the most prevalent cancers among women, with an estimated 660,000 new cases and 350,000 deaths worldwide in 2022.^1^ Its incidence and mortality both rank fourth among all cancers in women. In China, cervical cancer accounts for over 40% of all gynecological cancers, with approximately 110,000 new cases reported in 2022,^2^ posing a significant threat to women's health. Human papillomavirus (HPV) is a necessary but insufficient cause of cervical cancer, and HPV vaccination can reduce its incidence by around 70%.^3^, ^4^, ^5^ To eliminate cervical cancer as a public health problem by 2030, the World Health Organization (WHO) established global targets in 2020,^6^ which include the mandate that 90% of girls complete the full course of HPV vaccination by the age of 15. The HPV vaccine was first approved by the China Food and Drug Administration (CFDA) in July 2016,^7^ a decade after Merch's quadrivalent HPV vaccine, Gardasil, received its initial authorization.^8^ Although the HPV vaccination rate in China has increased from 0.01% in 2017 to 10.15% in 2022,^9^ it remains relatively low. Among girls aged 9-14, the one-dose coverage is only 4%,^9^ which is far from the target set by the WHO.

Vaccine coverage is influenced by both healthcare accessibility and public acceptance of vaccines.^10^ Socioeconomic indicators such as education, poverty, and insurance status largely capture disparities in accessibility and acceptance.^11^, ^12^ When sufficient policy and economic support is in place, it becomes particularly crucial to enhance public perceptions of vaccines and address vaccine hesitancy.^13^ Traditional investigations into vaccine confidence and hesitancy are primarily conducted through offline or online questionnaire surveys. In the Internet era, social media platforms serve as crucial channels for the public to disseminate and access health-related information, as well as to engage in discussions concerning public health matters.^14^, ^15^, ^16^ Additionally, media coverage and discourse significantly influence public attention toward public health issues, and some vaccine-related negative information is likely to undermine public acceptance of vaccines.^17^, ^18^ Several studies have analyzed the attitudes of the Chinese public towards the HPV vaccine by examining relevant posts on social platforms, which depicted the general public attitudes from an overall perspective yet without establishing correlations with the regional coverage rates of the HPV vaccine.^19^, ^20^ In China, there is significant variation in HPV vaccine coverage across different regions, with the one-dose coverage rate in some western provinces even falling below 5%.^9^ This constitutes one of the key challenges confronting China's national plan for cervical cancer elimination.

In this study, we aimed to investigate the Chinese public's attention to the HPV vaccine and determined whether vaccine discourse on Weibo was associated with regional HPV vaccine coverage in China. We examined HPV vaccine-related posts on the Weibo platform from two perspectives: discourse volume and discourse themes. We collected posts related to the HPV vaccine from Weibo, and each post was thematically annotated using a fine-tuned deep learning model. With over 500 million registered users, Weibo is one of the most popular social media platforms in China and serves as an important channel for public discussions on health-related topics.^21^, ^22^ Subsequently, we constructed multivariate regression models to explore the correlation between posts from different regions and vaccination coverage, while comparing these findings with conventional socioeconomic explanatory variables.

## 2 Methods

### 2.1 Data acquisition

We used “HPV vaccine” (in Chinese) as the keyword to search for posts on Weibo. The platform's retrieval algorithm automatically performed semantic expansion on keywords, returning posts that contain the original terms along with their approximate variants and related subject terms.^23^ A Python web crawler was employed to scrape the primary data of relevant Weibo posts, including the posting time, the main content of the post, as well as the user ID, IP geolocation, number of fans and gender of the publishers. In the primary analysis of this study, we collected relevant Weibo posts from January 2022 to December 2022. Since publicly available vaccination data are currently updated only through 2022, the Weibo data from this period were used to construct regional vaccine coverage interpretation models. Furthermore, we additionally collected data from the most recent one year period (January 2025 to December 2025) for comparison. Although the latest vaccination information is not available, the changes in public concerns reflected in discussions about the HPV vaccine are also of significant value for guiding vaccine promotion efforts.^19^ Especially with the gradual market launch of domestically produced HPV vaccines and the progressive approval of male vaccination in recent years,^24^, ^25^ it has become particularly important to promptly identify the foci of public concern. During the data preprocessing stage, duplicate posts published by the same publisher and posts without valid IP locations were removed. To improve data relevance and classification effectiveness, URLs and special characters were removed during text preprocessing.^26^ Long posts were subsequently segmented into shorter text chunks to facilitate downstream deep learning-based theme classification. Text segments that did not contain any keywords from the predefined domain-specific lexicon were considered irrelevant noise and excluded from further analysis.^27^, ^28^ The custom dictionary used was jointly compiled by two authors, containing a comprehensive range of keywords related to HPV. Data on HPV vaccine coverage by region were obtained from previously published articles,^9^ and this study reported HPV vaccination data for Chinese women aged 9-45 years using information from the China Immunization Information System (CIIS) electronic vaccine registry. The system covers all immunization clinics across China, recording information for every vaccine dose, including the vaccination date and the vaccine used. The rate we used was estimated cumulative coverage for one-dose and three-dose HPV vaccination among females aged 9–45 years. This was calculated by dividing the total number of females aged 9–45 years in a given study year who received one-dose or three-dose of the vaccine from 2017 (the time when the HPV vaccination was first available in China) to the study year, by the total number of females aged 9–45 years in the given study year.^9^ Other socioeconomic data were sourced from China's official statistical yearbooks and reports. Further details are available in the supplementary file.

### 2.2 Development of the annotation framework

The researchers designed an annotation framework by reviewing a sample of 1000 posts and drawing on existing literature that analyzes themes in HPV vaccine-related posts on social media.^20^, ^29^, ^30^ Our annotation framework covers the main themes related to HPV vaccine discussions on the Weibo platform, specifically: 1) expectations for vaccination, 2) vaccine-related policies and publicity, 3) experiences and feelings regarding vaccination, 4) issues in the vaccine market such as scalpers and smuggling, 5) difficulties in accessing the vaccine, and 6) popular science related to the vaccine. Detailed information regarding the annotation framework can be seen in Table S1.

### 2.3 Fine-tuning of deep learning model

Bidirectional Encoder Representation from Transformers (BERT), proposed by Google in 2018, is a “large” pre-trained natural language processing model that outperformed all models before it on the General Language Understanding Evaluation benchmark.^31^

Chinese-RoBERTa-wwm-ext is developed based on the RoBERTa architecture. The acronym “wwm” denotes Whole Word Masking, a technique involving the masking of entire words.^32^, ^33^ This approach enables the model to achieve a more robust comprehension of contextual nuances and semantic relationships, thereby enhancing the accuracy and performance of Chinese text processing tasks.

To deploy the model for analyzing Weibo posts related to HPV vaccines, the pre-trained Chinese RoBERTa-wwm-ext model was fine-tuned using our manually annotated dataset of Weibo posts, following the common paradigm of supervised fine-tuning for downstream text classification tasks. After two researchers (Wang ZW, and Li B) independently annotated the posts and compared their results, followed by a discussion with a third researcher (Wu JY) to resolve discrepancies, we manually annotated 2000 randomly selected posts. Given the relatively small number of posts annotated for Theme 4, we employed stratified sampling based on Theme 4, with 20% of the posts allocated to the test set and 80% used for model training. To enhance the stability and generalization ability of the model, we employed a five-fold cross-validation method and integrated the models using majority voting (based on the prediction results from more than half of the models to determine the annotation).^34^, ^35^ The Chinese-RoBERTa-wwm-ext model was used to predict annotations in the test set, and the model predictions were compared with manually generated human annotations. Human annotations were regarded as the ground truth (gold standard), which is a common practice in supervised machine learning and natural language processing studies.

*F*_*1*_ score and precision are widely used evaluation metrics in natural language processing and machine learning studies for assessing classification model performance. They are calculated as follows.

$$F_{1}\;score=2\;*\frac{1}{\frac{1}{precision}+\frac{1}{recall}}$$

Where,

$$precision=\frac{True\;Positive}{True\;Positive+False\;Positive}$$

$$recall=\frac{True\;Positive}{True\;Positive+False\;Negative}$$

### 2.4 Statistical analysis

We constructed multivariate regression models to examine the association between Weibo post volume and theme across different regions and vaccine coverage rates, and compared the results with models incorporating socioeconomic explanatory variables. All multivariable models were adjusted for HPV vaccine coverage prior to the study period to control for baseline vaccination levels. Since Theme 3-related posts originated from vaccinated individuals and Theme 4-related posts had an ambiguous relationship with the posters’ vaccination status, we constructed three models for any analyses based on post volume or themes. These models corresponded to three subsets: all posts, posts excluding Theme 3 and Theme 4, and posts excluding Theme 3. This approach allowed us to independently examine the association between potentially prospective posts and vaccine coverage. The number of Weibo posts from different regions was standardized according to the local internet user population. The socio-economic indicators under consideration include GDP (gross domestic product) per capita, health expenditure per capita, out-of-pocket share of health expenditure, proportion of population with tertiary education, unemployment rate, and number of community health service centers. We used a nested leave-one-out cross-validation (LOOCV) procedure in which variable selection was performed within each training fold. In each fold, candidate predictors were first screened by univariable analysis, and those with *P* < 0.1 were advanced to a multivariable model. We then iteratively removed the predictor with the highest variance inflation factor (VIF) until all retained predictors had VIF < 10. In addition, we conducted a sensitivity analysis using a more stringent VIF cutoff of 5. Retention frequency of each candidate predictor was recorded across all folds, and predictors retained in more than half of the folds were included in the final model. Model performance was quantified using the LOOCV *R*^*2*^, calculated from out-of-fold predictions. All analyses were conducted in R.4.3.3 (R Core Team, Vienna, Austria).

## 3 Results

A total of 61,384 posts were retrieved using “HPV vaccine” as the search keyword during the period from January 1, 2022, to December 31, 2022. After a preliminary review of the content from the scraped posts, we identified a series of homogeneous posts originating from fans of an idol. These posts contain the hashtag “HPV vaccine”, but the content was entirely unrelated to the vaccine. Consequently, they were excluded from the dataset (N = 8603). For posts with identical content posted by the same poster, only the initial post was retained, while the others were excluded (N = 1074). Posts from users with hidden IP addresses and those originating from countries or regions outside our target 31 provinces were excluded (N = 18,505). Additionally, after filtering the text segments using a custom dictionary, 3564 posts containing no valid content were removed. Finally, 29,638 out of the original 61,384 posts were retained, originating from across 31 provinces (Fig. S1). The fine-tuned model achieved a macro F_1_ score of 0.8530 and a micro F_1_ score of 0.8562, indicating effective classification capability and balanced performance across all categories. In the test set, when evaluated against six categories annotation framework, the deep learning model achieved a precision ranging from 79.7% to 90.2%. The detailed performance of the model on each theme can be found in Table 1.

**Table 1:** Theme annotation categories for posts related to “HPV Vaccine” on Weibo platform, January 2022 to December 2022.

Annotation category^a^	Definition	Performance of ensemble deep learning model^b^	
		F_1_-score	Precision
Theme1	This post expresses the expectation for HPV vaccination.	0.837	0.797
Theme2	This post refers to the policies, programmes, or publicity related to HPV vaccination.	0.895	0.902
Theme3	This post refers to the experiences and feelings about HPV vaccination, including side effects.	0.846	0.897
Theme4	This post refers to the chaos in the HPV vaccine market, such as scalpers and smuggling.	0.865	0.889
Theme5	This post is related to HPV vaccine access, including issues such as lottery systems, long waiting times, and high costs.	0.815	0.846
Theme6	This post refers to the dissemination of knowledge related to the HPV vaccine.	0.861	0.840

HPV: human papillomavirus.

a Each post can be annotated with multiple themes or not belong to any theme.

b Performance of ensemble deep learning models was assessed by comparing their annotations with annotations made by humans, with human annotation as the gold standard.

Overall, discussions regarding the HPV vaccine on the Weibo platform were predominantly centered on personal experiences and feelings regarding HPV vaccination (Theme 3), with 30.69% (9096/29638) of posts being related to this theme. In addition, those related to other themes included vaccination policy and publicity (Theme 2, 27.75%), popular science on vaccine-related knowledge (Theme 6, 19.37%), expectations for vaccination (Theme 1, 12.92%), complaints about long queues and high costs (Theme 5, 10.57%), and concerns about the vaccine market (Theme 4, 5.74%). The coverage of HPV vaccines in China exhibits a pronounced regional imbalance, generally following a trend of higher coverage in the eastern regions and lower coverage in the western regions. This disparity in vaccine coverage is also echoed by discussions about HPV on social media platforms (Fig. 1A and B). In provinces with leading vaccination rates, such as Beijing and Shanghai, knowledge popularization related to vaccines constitutes the most prevalent theme on the Weibo platform. In contrast, provinces with lower vaccination coverage tend to focus more on Theme 2 and Theme 3 (Fig. 2).

**Fig. 1:**
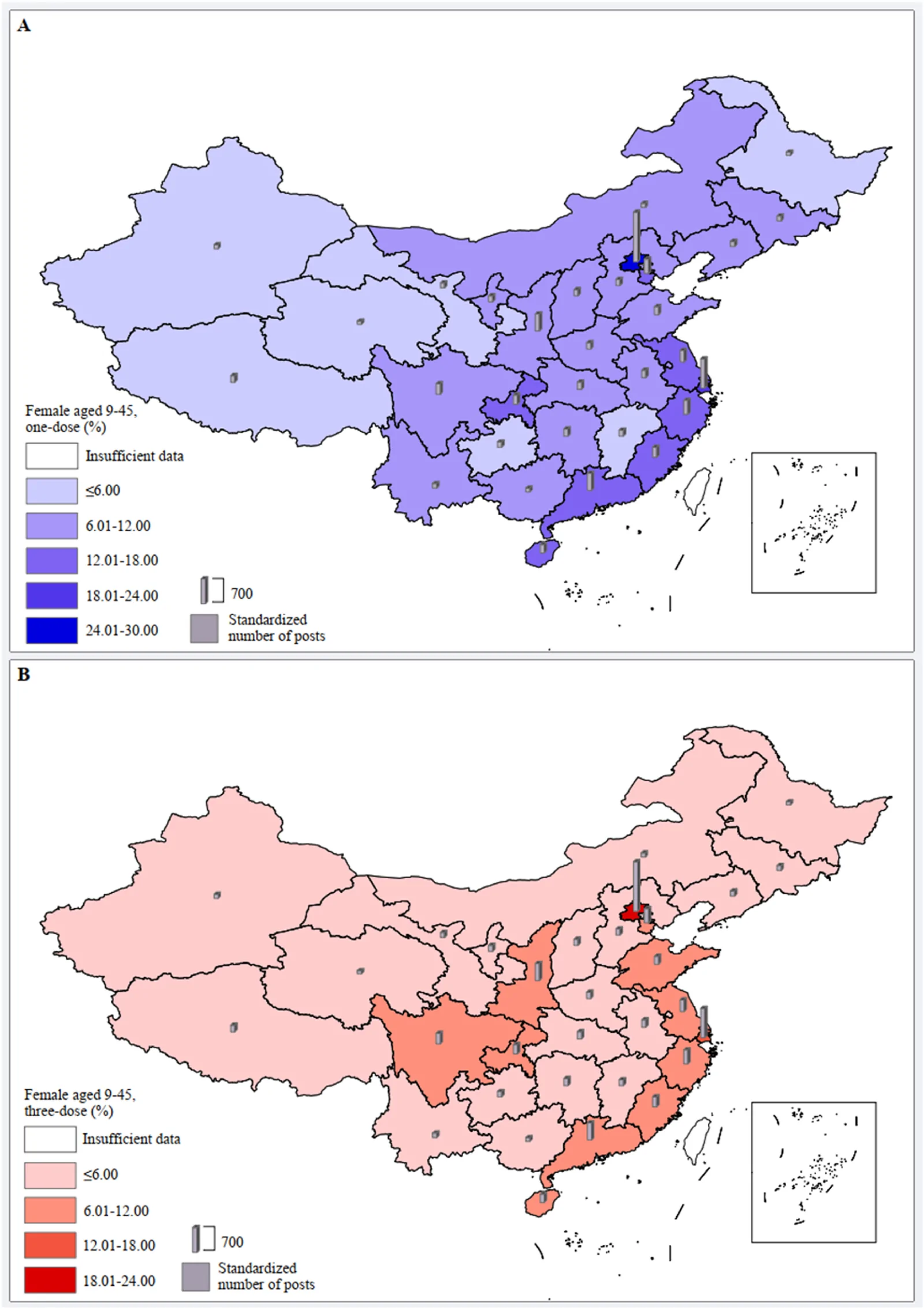
Number of HPV Vaccine-Related Weibo posts per region (January 2022 to December 2022) and HPV vaccine (A: one-dose, B: three-dose) coverage rate among women aged 9–45 years in 2022.

**Fig. 2:**
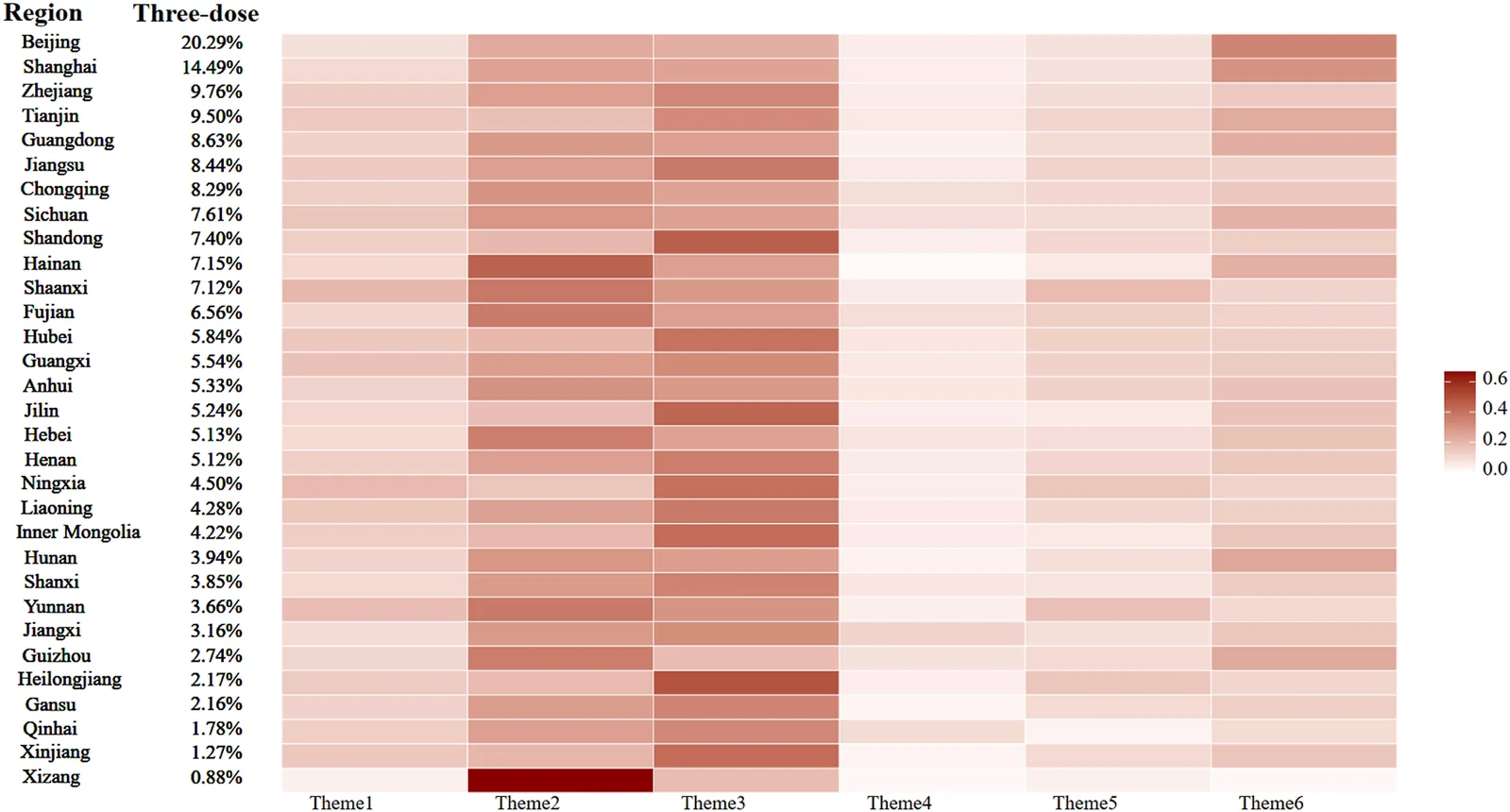
Proportion of Weibo posts related to each theme, by region.

In the univariate analysis, regardless of the theme, the number of vaccine-related posts exhibited a significant correlation with regional vaccine coverage. Moreover, both the one-dose rate and the three-dose rate (one-dose: *r* = 0.869, 95% confidence interval (CI) [0.744, 0.936], *P* < 0.001; three-dose: *r* = 0.920 [0.840, 0.961], *P* < 0.001) showed the strong correlation with the total number of posts, and this correlation was stronger than that observed for any socioeconomic factor (Table 2). For themes, the volume of posts related to Theme 3 exhibited the strongest correlation with vaccination coverage (one-dose: *r* = 0.895 [0.792, 0.949], *P* < 0.001; three-dose: *r* = 0.942 [0.883, 0.972], *P* < 0.001). Upon excluding Theme 3, or both Theme 3 and Theme 4, the correlation between the volume of potentially prospective posts and vaccine coverage decreased to some extent, and its association with one-dose coverage was no longer stronger than that with per-capita GDP. Details of the univariate models are presented in Supplementary Table S2. Results of the multivariate models are shown in Table 3. Similar to the findings from univariate analysis, the model constructed based on post volume better out-of-sample model performance for regional coverage across total Weibo posts compared with the model built on socioeconomic factors (LOOCV *R*^*2*^:one-dose 0.643 vs 0.606; three-dose 0.818 vs 0.662). However, when potentially prospective posts are considered, the generalizability of the model deteriorates, and it no longer exhibits advantages for the one-dose vaccination (Table 3). It is worth noting that, in the full model accounting for both post-related variables and socioeconomic factors, model performance improved after excluding Theme 3 and Theme 4 (LOOCV *R*^*2*^:one-dose 0.620 vs 0.593; three-dose 0.797 vs 0.720), and a similar result was observed when only Theme 3 was excluded (LOOCV *R*^*2*^:one-dose 0.608 vs 0.593; three-dose 0.734 vs 0.720). Furthermore, after excluding Theme 3 and Theme 4, the full model constructed with potentially prospective posts and socioeconomic factors (LOOCV *R*^*2*^: one-dose = 0.620; three-dose = 0.797) outperformed the other three models, which were built using socioeconomic factors (LOOCV *R*^*2*^: one-dose = 0.606; three-dose = 0.662), post-count-related variables (LOOCV *R*^*2*^: one-dose = 0.461; three-dose = 0.789), and topic-specific post variables (LOOCV *R*^*2*^: one-dose = 0.604; three-dose = 0.786), respectively.

**Table 2:** Correlation of provincial HPV vaccination coverage with socioeconomic factors and Weibo posts.

Variable		Female aged 9–45, one-dose (%)		Female aged 9–45, three-dose (%)	
		Correlation (95%CI)	*P*	Correlation(95%CI)	*P*
***Socioeconomic factors***					
GDP per capita (in 10,000 CNY)		0.849 (0.708, 0.925)	<0.001	0.865 (0.736, 0.933)	<0.001
Health expenditure per capita (in 1000 CNY)		0.730 (0.506, 0.861)	<0.001	0.798 (0.619, 0.898)	<0.001
Out-of-pocket share of health expenditure (%)		−0.353 (−0.628, 0.002)	0.052	−0.364 (−0.636, −0.011)	0.044
Proportion of population with tertiary education (%)		0.721 (0.492, 0.857)	<0.001	0.817 (0.651, 0.908)	<0.001
Unemployment rate (%)		−0.003 (−0.357, 0.351)	0.985	0.013 (−0.343, 0.366)	0.943
Number of community health service centers (per 100,000 population)		0.386 (0.037, 0.651)	0.032	0.473 (0.143, 0.709)	0.007
***Weibo post***s a					
Number of posts	total	0.869 (0.744, 0.936)	<0.001	0.920 (0.840, 0.961)	<0.001
	exclude theme3 and theme4	0.848 (0.706, 0.925)	<0.001	0.899 (0.799, 0.950)	<0.001
	exclude theme3	0.852 (0.712, 0.926)	<0.001	0.903 (0.807, 0.953)	<0.001
The proportion of posts from females	total	−0.526 (−0.742, −0.211)	0.002	−0.515 (−0.735, −0.196)	0.003
	exclude theme3 and theme4	−0.441 (−0.688, −0.103)	0.013	−0.437 (−0.685, −0.097)	0.014
	exclude theme3	−0.445 (−0.690, −0.107)	0.012	−0.442 (−0.689, −0.104)	0.013
Number of posts from influential users^b^	total	0.810 (0.638, 0.904)	<0.001	0.881 (0.766, 0.942)	<0.001
	exclude theme3 and theme4	0.800 (0.621, 0.899)	<0.001	0.872 (0.750, 0.937)	<0.001
	exclude theme3	0.803 (0.627, 0.901)	<0.001	0.875 (0.755, 0.939)	<0.001
Number of posts annotated as theme1		0.873 (0.752, 0.938)	<0.001	0.900 (0.800, 0.951)	<0.001
Number of posts annotated as theme2		0.807 (0.635, 0.903)	<0.001	0.811 (0.641, 0.905)	<0.001
Number of posts annotated as theme3		0.895 (0.792, 0.949)	<0.001	0.942 (0.883, 0.972)	<0.001
Number of posts annotated as theme4		0.831 (0.675, 0.916)	<0.001	0.891 (0.784, 0.946)	<0.001
Number of posts annotated as theme5		0.837 (0.686, 0.919)	<0.001	0.865 (0.737, 0.933)	<0.001
Number of posts annotated as theme6		0.806 (0.632, 0.903)	<0.001	0.882 (0.767, 0.942)	<0.001

HPV: human papillomavirus.

a All post-count variables were standardized by the provincial total number of internet users in 2022.

b Influential user is defined as user with more than 10,000 followers.

**Table 3:** Comparison of multivariate linear regression models for factors associated with HPV vaccination coverage among females aged 9–45 years in 2022.

Models^a^		Number of candidate factors	Factors used^b^	*P*^c^	LOOCV R^2^	ΔLOOCV R^2^
***Female aged 9–45, one-dose (%)***						
Coverage rate in 2021		/	/	5.787*10^−19^	0.606	/
Socioeconomic factors		6	4	3.821*10^−17^	0.606	0.000
All posts^d^, posts from influential users and the proportion from females	total	3	2	8.191*10^−17^	0.643	0.037
	exclude theme3 and theme4	3	2	1.027*10^−16^	0.604	−0.002
	exclude theme3	3	2	1.094*10^−16^	0.607	0.001
Number of posts annotated as each theme	total	6	4	8.252*10^−17^	0.478	−0.128
	exclude theme3 and theme4	4	3	5.576*10^−18^	0.461	−0.145
	exclude theme3	5	4	8.513*10^−17^	0.430	−0.176
All factors	total	15	7	9.663*10^−15^	0.593	−0.013
	exclude theme3 and theme4	13	6	4.472*10^−15^	0.620	0.014
	exclude theme3	14	7	9.219*10^−15^	0.608	0.002
***Female aged 9–45, three-dose (%)***						
Coverage rate in 2021		/	/	3.511*10^−20^	0.606	/
Socioeconomic factors		6	4	2.462*10^−18^	0.662	0.056
All posts, posts from influential users and the proportion from females	total	3	2	6.805*10^−18^	0.818	0.212
	exclude theme3 and theme4	3	2	5.541*10^−18^	0.786	0.180
	exclude theme3	3	2	5.278*10^−18^	0.793	0.187
Number of posts annotated as each theme	total	6	4	1.630*10^−17^	0.706	0.100
	exclude theme3 and theme4	4	3	2.134*10^−18^	0.679	0.073
	exclude theme3	5	4	1.990*10^−17^	0.701	0.095
All factors	total	15	7	2.718*10^−16^	0.720	0.114
	exclude theme3 and theme4	13	6	2.566*10^−16^	0.797	0.191
	exclude theme3	14	7	3.020*10^−16^	0.734	0.128

HPV: human papillomavirus.

a All models have adjusted the coverage rate for the year 2021, and the summary of each model is available in the supplementary materials.

b Variables retained in more than half of all folds were included in the final model; the retention frequency of each variable is provided in the supplementary materials.

c The *P* values refer to the overall F test of the final multivariable linear model fitted on the full dataset after LOOCV-based variable selection.

d All post-count variables were standardized by the provincial total number of internet users in 2022.

In the fully-adjusted multivariate models incorporating socioeconomic factors and potentially prospective posts (exclude Theme3, or both Theme3 and Theme4), the direction of association between certain socioeconomic variables and coverage in the full model might be opposite to that observed in the univariable analyses (for the out-of-pocket share of health expenditure, the proportion of population with tertiary education and the number of community health service centers; Table 2, Table 4). Comparable findings were observed even in the sensitivity analysis using a stricter VIF cutoff of 5 (for the number of community health service centers, and out-of-pocket share of health expenditure; Table 2 and Table S5). In addition, for variables related to posts, only a significant association between the number of Theme 1 related posts and three-dose coverage (*β* = 0.027 [0.003, 0.052], *P* = 0.028) was observed in the full model excluding Theme 3.

**Table 4:** Models of HPV vaccination coverage for Weibo posts and socioeconomic factors in 31 provinces in China.

Full models	Variable^a^	Female aged 9–45, one-dose (%)		Female aged 9–45, three-dose (%)	
		Regression coefficient (95% CI)	*P*	Regression coefficient (95% CI)	*P*
**Exclude theme3 and theme4**	GDP per capita (in 10,000 CNY)	0.275 (−0.004, 0.554)	0.053	0.155 (−0.026, 0.335)	0.090
	Out-of-pocket share of health expenditure (%)	0.008 (−0.114, 0.130)	0.894	0.131 (0.053, 0.208)	**0.002**
	Proportion of population with tertiary education (%)	−0.219 (−0.404, −0.033)	**0.023**	−0.031 (−0.154, 0.093)	0.611
	Number of community health service centers (per 100,000 population)	0.127 (−1.870, 2.123)	0.897	−1.069 (−2.369, 0.231)	0.102
	The proportion of posts from females (%)	−0.002 (−0.049, 0.044)	0.921	−0.002 (−0.032, 0.029)	0.919
	Number of posts^b^ annotated as theme6	−0.004 (−0.019, 0.011)	0.620	0.005 (−0.006, 0.015)	0.360
	Coverage rate in 2021	1.590 (1.237, 1.943)	**<0.001**	1.402 (1.042, 1.762)	**<0.001**
**Exclude theme3**	GDP per capita (in 10,000 CNY)	0.307 (0.048, 0.566)	**0.022**	0.092 (−0.073, 0.257)	0.259
	Out-of-pocket share of health expenditure (%)	0.016 (−0.088, 0.119)	0.759	0.101 (0.032, 0.171)	**0.006**
	Proportion of population with tertiary education (%)	−0.255 (−0.419, −0.090)	**0.004**	−0.041 (−0.148, 0.067)	0.441
	Number of community health service centers (per 100,000 population)	0.152 (−1.760, 2.067)	0.870	−0.647 (−1.892, 0.599)	0.293
	The proportion of posts from females (%)	0.000 (−0.047, 0.048)	0.983	−0.005 (−0.035, 0.026)	0.753
	Number of posts annotated as theme1	0.034 (−0.006, 0.074)	0.093	0.027 (0.003, 0.052)	**0.030**
	Number of posts annotated as theme4	−0.045 (−0.117, 0.027)	0.208	0.000 (−0.048, 0.048)	0.995
	Coverage rate in 2021	1.486 (1.165, 1.808)	**<0.001**	1.362 (1.058, 1.665)	**<0.001**

HPV: human papillomavirus, CI: confidence interval.

a As presented in Table 3, the selection process resulted in variables from the initial candidate factors, and the coverage rate in 2021 was controlled.

b All post-count variables were standardized by the provincial total number of internet users in 2022.

From January 2025 to December 2025, a total of 31,865 original posts were retrieved, representing a decrease of 48.1% compared to three years earlier (from Janrary 2022 to December 2022). Among them, the proportions of posts from male users (54.24% vs. 35.20%, *χ*^*2*^ = 3126.6, *P* < 0.001) and from influential users (50.84% vs. 37.19%, *χ*^*2*^ = 1606.8, *P* < 0.001) both increased significantly. After screening, a total of 21,767 posts were included for theme annotation using the deep learning model (Fig. S1). Except for the number of posts related to Theme 6, which increased compared to three years earlier, the numbers of posts related to the other themes all decreased to varying degrees (Fig. 3). Among them, the decline in posts related to Theme 1 was the most pronounced, amounting to only 7.86% of the level observed three years ago.

**Fig. 3:**
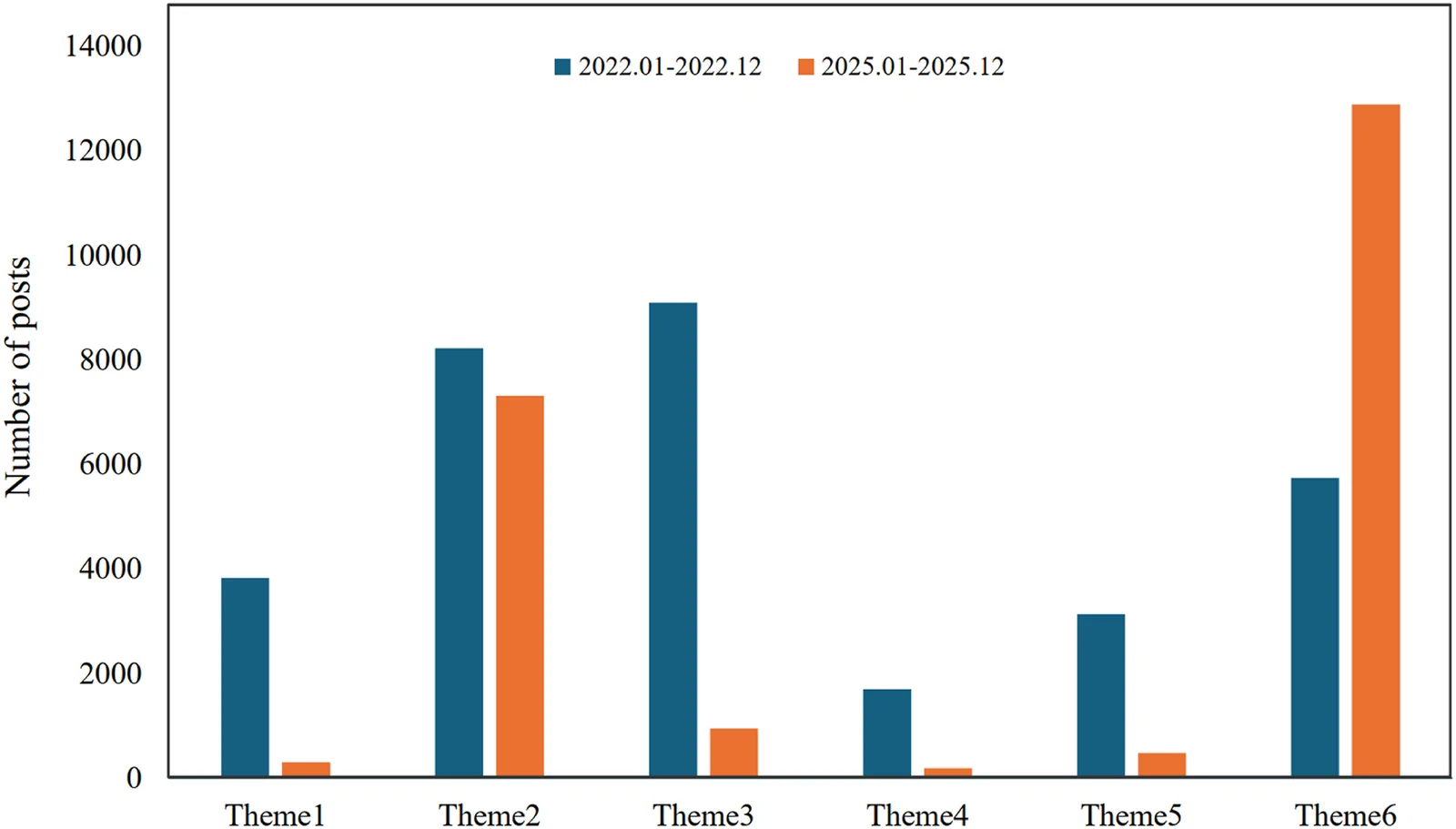
Comparison of HPV vaccine-related posts on the weibo platform in 2022 and 2025.

## 4 Discussion

In this study, we leveraged a large-scale social media dataset from Weibo and a fine-tuned deep learning model to systematically examine the relationship between public discourse on the HPV vaccine and regional vaccination coverage in China. Our findings suggest that HPV vaccine-related discourse on Weibo was associated with regional HPV vaccine coverage in China, especially for three-dose coverage. However, the association differed by discourse theme and by vaccination outcome, requiring cautious interpretation.

Posts describing vaccination experiences and feelings are generated after vaccination. Their strong correlation with coverage is expected and should not be interpreted as prospective signal for vaccination or evidence that experience-sharing drives vaccination. In contrast, theme 1, themes 5 and 6 are more directly prospective in nature, whereas may capture unmet needs or informational demand. When analyses distinguished between these types of themes, the association with coverage persisted but became more modest. This suggests that overall post volume may mix anticipatory signals with retrospective accounts of vaccination activity; theme-specific measures are therefore more informative for interpreting the nature of the association and identifying potentially actionable discourse, even though aggregate volume may still show a stronger out-of-sample association with coverage. The dose-specific pattern also merit attention. For three-dose coverage, theme-based variables showed greater incremental value, whereas they showed poorer performance for one-dose coverage. One possible explanation is that the initiation of the first dose is more affected by price, affordability and availability, whereas completion of the full vaccination schedule may depend more on sustained attention and demand.^36^ Social media discourse may be more informative about the broader vaccination environment associated with completion of the multi-dose schedule than about initial vaccine uptake.

Although the individual coefficients of some socioeconomic variables were unstable in the full model, likely due to collinearity and limited statistical power, when socioeconomic factors were combined with potentially prospective posts variables, model performance was consistently improved compared with models constructed using either set of factors alone. This pattern supports the view that social media discourse may capture a different dimension of the vaccination environment—public attention, perceived barriers, and informational needs—that structural indicators do not fully measure. Socioeconomic indicators capture structural capacity, whereas social media discourse may capture the population's perceived and communicative response to that environment.^37^ Their complementary explanatory performance suggests that digital discourse may provide information that is not fully represented by conventional socioeconomic indicators. A recent review describes the dual role of internet-based information and social media: they may increase HPV vaccine awareness and demand, but can also amplify misinformation, hesitancy, and access-related concerns.^38^ Social media surveillance should not be viewed as a replacement for traditional structural indicators but rather as a real-time, high-resolution lens for understanding the psychosocial and informational barriers that persist even when economic and infrastructural conditions are partially met.^39^

Geographically, provinces with higher vaccination coverage, such as Beijing and Shanghai, exhibited a greater proportion of posts related to vaccine science popularization, whereas provinces with lower coverage tended to focus more on policies, programmes, and publicity. This may reflect different stages of vaccine rollout. Where cost and access remain limiting, discourse should address policy support, and special offers. Where access is less constrained, discourse may need to sustain demand, promote completion of the full schedule, and counter misinformation. The increasing participation of male posters and influential posters suggests that HPV vaccination is becoming framed as a broader public health issue and concerns all genders. In recent years, a variety of affordable domestic vaccines have become available.^40^ China has also recently approved HPV vaccination for males. These will bring a great shift in the vaccination landscape.

A reduction in the total number of posts and shifts in their composition reflect a decline in public discussions regarding HPV vaccine on social media. Such discussions have become more focused on vaccine policies and the exchange of vaccine-related knowledge, whereas communication concerning expectations for vaccines, sharing of vaccination experiences, and vaccination-related difficulties have decreased substantially. These may partly reflect the shrinking pool of unvaccinated eligible individuals, improved vaccine availability, or the gradual normalization of HPV vaccination. As coverage data for the later period were unavailable, we could not determine whether these discourse changes precede or predict changes in coverage.

These findings support a cautious role for social media monitoring in public health planning. Social media data may help identify region-specific concerns, unmet demand, access barriers, and informational gaps in near real time. However, they should not be used as a stand-alone measure of vaccine confidence or as a replacement for surveys, and structural indicators. The strengths of social-media monitoring lie in its real-time performance and operability, offering a convenient window to understand public attitudes toward vaccination.

Previous studies have utilized Twitter post data to predict county-level heart disease mortality,^41^ as well as investigated the association between exposure to Twitter information and HPV vaccine coverage.^42^, ^43^ However, these studies were conducted in developed Western countries. China, as a developing country with substantial geographical disparities, still has a HPV vaccine coverage that lags far behind the global average. Our findings support a cautious but potentially useful role for social media monitoring in HPV vaccination surveillance. The main value of such data may not be to replace conventional vaccination coverage surveillance or survey-based measures of vaccine confidence. Instead, social media can provide a relatively timely source of information about what people are discussing, which barriers are salient, and how vaccination-related information needs differ across regions. Our study carries certain public health implications, demonstrating that region-level, theme-specific social media discussion content serves as a supplementary indicator for understanding regional disparities in HPV vaccine coverage in China.

This study has several limitations. First, the ecological and observational design precludes causal inference. The observed associations may reflect vaccination generating social media discussion, social media exposure affecting vaccination behavior, shared underlying determinants, or combinations of these pathways. Second, although nested leave-one-out cross-validation was used, the small number of units limits statistical power and the stability and generalizability of model estimates; the findings and differences in model performance should therefore be regarded as exploratory. Third, Weibo users are not representative of the entire population and may be younger and more urban, potentially limiting generalizability. Fourth, IP-based geographic information may not accurately represent the user's residence or vaccination location. Fifth, post counts were standardized by the provincial internet-user population rather than the population eligible for HPV vaccination, which may affect comparisons across regions. Next, of the socioeconomic indicators, demographic metrics come from the 2020 census, whereas unemployment rates rely on 2021, leading to potential temporal mismatches. Finally, the absence of vaccination coverage data after 2022 prevented us from determining whether changes in discourse between 2022 and 2025 preceded subsequent changes in vaccination coverage. Consequently, the present study should be viewed as evidence of regional association and potential surveillance utility rather than validation of social media as a temporal leading indicator of vaccination uptake.

In conclusion, HPV vaccine-related discourse on Weibo was strongly associated with regional HPV vaccination coverage in China, particularly for three-dose coverage. Social media discourse reflects the regional vaccination environment, but the meaning of this association differs according to whether discourse is retrospective or potentially prospective. After accounting for retrospective discourse, potentially prospective social media signals retained complementary information when considered alongside socioeconomic factors. These findings suggest that social media may provide a useful supplementary lens for understanding regional differences in public attention, information needs, and barriers related to HPV vaccination. Future longitudinal studies integrating temporally aligned social media data, vaccination records, and individual-level survey data are needed to determine whether theme-specific social media signals can reliably anticipate changes in HPV vaccination behavior.

## CRediT authorship contribution statement

**Zhongwen Wang:** Writing – review & editing, Writing – original draft, Visualization, Methodology, Data curation. **Xinsheng Wu:** Writing – review & editing, Writing – original draft, Visualization, Methodology. **Jinyuan Wu:** Writing – review & editing, Writing – original draft, Data curation. **Guozhen Wu:** Writing – review & editing, Writing – original draft, Methodology, Data curation. **Bin Li:** Writing – review & editing, Data curation. **Huachun Zou:** Writing – review & editing, Funding acquisition, Conceptualization.

## Statement regarding the use of AI

During the preparation of this work the authors used DeepSeek in order to improve language and readability of the manuscript. All outputs were carefully reviewed and revised by the authors, who take full responsibility for the final content of the manuscript.

## Funding

This study was supported by the Natural Science Foundation of China General Program (8257121609), the Prevention and Control of Emerging and Major Infectious Diseases-National Science and Technology Major Project (2025ZD01905200), and the National Key Technologies R&D Program of China (2023YFC2306700). All funding parties did not have any role in the design of the study or in the explanation of the data.

## Declaration of competing interest

The authors declare that they have no known competing financial interests or personal relationships that could have appeared to influence the work reported in this paper.

## Data Availability

Data will be made available on request.

## Abbreviations:

BERT: Bidirectional Encoder Representation from Transformers
CI: confidence interval
CFDA: China Food and Drug Administration
CIIS: China Immunization Information System
HPV: human papillomavirus
LOOCV: leave-one-out cross-validation
WHO: World Health Organization
VIF: variance inflation factor
