# Supplementary material for: Association between HPV vaccine-related social media discourse and regional HPV vaccine coverage in China
Source: J Virus Erad. 2026 Oct 29;12(4):100639. doi: 10.1016/j.jve.2026.100639 (PMC13763425; doi:10.1016/j.jve.2026.100639)
Supplement: Supplementary file 1 [file mmc1.docx]

Supplemental materials for:

**Association between HPV vaccine-related social media discourse and regional HPV vaccine coverage in China**

**Content**

[**Table S1. Examples of Weibo posts and their annotation categories.**	2](#_Toc240593669)

[**About the Chinese-RoBERTa-wwm-ext deep learning model.**	3](#_Toc240593670)

[**Table S2. Details of all univariate models.**	4](#_Toc240593671)

[**Table S3. Details of multivariate models.**	5](#_Toc240593672)

[**Table S4. The retention frequency of candidate factors.**	7](#_Toc240593673)

[**Figure S1. Flowchart of Weibo posts screening.**	9](#_Toc240593674)

[**Table S5. Sensitivity analysis (VIF cutoff of 5) for the model of HPV vaccination coverage for Weibo posts and socioeconomic factors in 31 provinces in China.**	10](#_Toc240593675)

[**Table S6. Data on HPV vaccine coverage and socioeconomic characteristics across regions.**	11](#_Toc240593676)

[**Table S7. A Custom Lexicon for Long Text Segmentation in Weibo Posts (in Chinese).**	13](#_Toc240593677)

| **Table S1. Examples of Weibo posts and their annotation categories.** | |
| --- | --- |
| Weibo post | Annotation ^a^ |
| I must get my first dose of the HPV vaccine before the end of May. Please!! | Theme1 |
| #Experts remind age-eligible women to get HPV vaccine as early as possible# You think I don’t want to?! I can’t get an appointment!!! | Theme1, Theme2,  Theme5 |
| The first dose of the HPV vaccine has been gotten—quite expensive and painful though. | Theme3,  Theme5 |
| #Woman scammed out of 12,000 CNY by scalper while booking HPV vaccine# Luckily, I finished my HPV vaccination two years ago. I wouldn't even have known about HPV vaccine if my friend hadn't told me about it. Later, she asked me to get vaccinated together. It's really better to get the vaccine as early as possible for peace of mind. | Theme3,  Theme4 |
| #Developing anxiety about the HPV vaccine# I’ve been booking it for a long time, hoping to get it soon. | Theme1,  Theme5 |
| Getting HPV vaccination and undergoing regular cervical cancer screenings are effective measures to prevent cervical cancer. Eligible women should get vaccinated as early as possible. | Theme2,  Theme6 |
| 【XX Pharmaceutical Company】In-depth analysis of HPV vaccine industry: Vast market potential with accelerating domestic penetration rate. | This post does not belong to any theme. |

^a^ Definition of every theme is shown in Table 1.

# **About the Chinese-RoBERTa-wwm-ext deep learning model.**

Bidirectional Encoder Representation from Transformer (BERT), proposed by Google in 2018, is a “large” pre-trained natural language processing model that outperformed all models before it on the General Language Understanding Evaluation benchmark.

Chinese-RoBERTa-wwm-ext is developed based on the RoBERTa architecture. The acronym “wwm” denotes Whole Word Masking, a technique involving the masking of entire words. This approach enables the model to achieve a more robust comprehension of contextual nuances and semantic relationships, thereby enhancing the accuracy and performance of Chinese text processing tasks.

Compared to the original BERT model, Chinese-RoBERTa-wwm-ext incorporates adjustments in training data scale and training steps to further enhance model performance and robustness. Moreover, it was pre-trained on a large-scale unsupervised corpus, enabling it to possess robust capabilities in language understanding and generation. This model can be widely applied to various natural language processing tasks, such as text classification, named entity recognition, and sentiment analysis. Utilizing this model as a foundation, fine-tuning and transfer learning on different downstream tasks can achieve more accurate and efficient processing of Chinese text.

Huggingface address: <https://huggingface.co/hfl/chinese-roberta-wwm-ext>

| **Table S2. Details of univariate models.** | | | | | |
| --- | --- | --- | --- | --- | --- |
| **Models** | | **Female aged 9–45,**  **one-dose (%)** | | **Female aged 9–45,**  **three-dose (%)** | |
|  |  | **Regression coefficient (95% CI)** | ***P*** | **Regression coefficient (95% CI)** | ***P*** |
| Coverage rate in 2021 | | 1.417 (1.278, 1.557) | <0.001 | 1.478 (1.347, 1.609) | <0.001 |
| GDP per capita (in 10,000 CNY) | | 1.233 (0.942, 1.524) | <0.001 | 0.947 (0.738, 1.156) | <0.001 |
| Health expenditure per capita (in 10,00 CNY) | | 1.320 (0.851, 1.790) | <0.001 | 1.089 (0.777, 1.402) | <0.001 |
| Out-of-pocket share of health expenditure (%) | | -0.369 (-0.742, 0.003) | 0.052 | -0.287 (-0.567, -0.008) | 0.044 |
| Proportion of population with tertiary education (%) | | 0.569 (0.361, 0.776) | <0.001 | 0.486 (0.356, 0.616) | <0.001 |
| Unemployment rate (%) | | -0.027 (-3.065, 3.011) | 0.985 | 0.081 (-2.210, 2.372) | 0.943 |
| Number of community health service centers (per 100,000 population) | | 6.118 (0.563, 11.674) | 0.032 | 5.685 (1.657, 9.660) | 0.007 |
| Number of posts ^a^ | total | 0.018 (0.014, 0.021) | <0.001 | 0.014 (0.012, 0.016) | <0.001 |
|  | exclude theme3 and theme4 | 0.024 (0.018, 0.029) | <0.001 | 0.019 (0.015, 0.022) | <0.001 |
|  | exclude theme3 | 0.022 (0.017, 0.027) | <0.001 | 0.018 (0.015, 0.021) | <0.001 |
| The proportion of posts from females (%) | total | -0.357 (-0.577 -0.138) | 0.002 | -0.264 (-0.430, -0.097) | 0.003 |
|  | exclude theme3 and theme4 | -0.208 (-0.368 -0.047) | 0.013 | -0.155 (-0.276, -0.034) | 0.014 |
|  | exclude theme3 | -0.225 (-0.398 -0.053) | 0.012 | -0.169 (-0.299, -0.039) | 0.013 |
| Number of posts from influential users ^b^ | total | 0.032 (0.023, 0.040) | <0.001 | 0.026 (0.021, 0.031) | <0.001 |
|  | exclude theme3 and theme4 | 0.035 (0.025, 0.045) | <0.001 | 0.029 (0.022, 0.035) | <0.001 |
|  | exclude theme3 | 0.033 (0.024, 0.042) | <0.001 | 0.027 (0.021, 0.033) | <0.001 |
| Number of posts annotated as theme1 | | 0.179 (0.141, 0.217) | <0.001 | 0.139 (0.114, 0.165) | <0.001 |
| Number of posts annotated as theme2 | | 0.063 (0.046, 0.081) | <0.001 | 0.048 (0.035, 0.061) | <0.001 |
| Number of posts annotated as theme3 | | 0.079 (0.064, 0.094) | <0.001 | 0.063 (0.054, 0.071) | <0.001 |
| Number of posts annotated as theme4 | | 0.316 (0.235, 0.396) | <0.001 | 0.255 (0.206, 0.305) | <0.001 |
| Number of posts annotated as theme5 | | 0.187 (0.140, 0.233) | <0.001 | 0.145 (0.113, 0.177) | <0.001 |
| Number of posts annotated as theme6 | | 0.046 (0.033, 0.059) | <0.001 | 0.038 (0.031, 0.046) | <0.001 |
| CI: confidence interval  ^a^ All post‑count variables were standardized by the provincial number of internet users.  ^b^ Influential user is defined as user with more than 10,000 followers. | | | | | |

| **Table S3. Details of multivariate models.** | | | | | | |
| --- | --- | --- | --- | --- | --- | --- |
| **Model** | | **Variable** | **Female aged 9–45,**  **one-dose (%)** | | **Female aged 9–45,**  **three-doses (%)** | |
|  |  |  | **Regression coefficient (95% CI)** | ***P*** | **Regression coefficient (95% CI)** | ***P*** |
| ***Socioeconomic factors*** | | GDP per capita (in 10,000 CNY) | 0.297 (0.044, 0.550) | **0.023** | 0.131 (-0.035, 0.298) | 0.117 |
|  |  | Out-of-pocket share of health expenditure (%) | 0.023 (-0.075, 0.120) | 0.636 | 0.122 (0.056, 0.187) | **0.001** |
|  |  | Proportion of population with tertiary education (%) | -0.239 (-0.397, -0.082) | **0.004** | -0.008 (-0.117, 0.100) | 0.874 |
|  |  | Number of community health service centers (per 100,000 population) | 0.033 (-1.844, 1.910) | 0.972 | -1.023 (-2.275, 0.230) | 0.105 |
|  |  | Coverage rate in 2021 | 1.536 (1.285, 1.787) | **<0.001** | 1.520 (1.266, 1.773) | **<0.001** |
| ***All posts ^a^, posts from influential users ^b^ and the proportion from females*** | total | Number of posts | -0.003 (-0.008, 0.002) | 0.226 | 0.000 (-0.003, 0.004) | 0.816 |
|  |  | The proportion of posts from females (%) | -0.031 (-0.105, 0.044) | 0.410 | -0.035 (-0.085, 0.015) | 0.165 |
|  |  | Coverage rate in 2021 | 1.584 (1.212, 1.956) | **<0.001** | 1.387 (0.992, 1.782) | **<0.001** |
|  | exclude theme3 and theme4 | Number of posts | -0.003 (-0.010, 0.003) | 0.263 | 0.000 (-0.005, 0.005) | 0.979 |
|  |  | The proportion of posts from females (%) | -0.014 (-0.064, 0.036) | 0.571 | -0.026 (-0.059, 0.007) | 0.124 |
|  |  | Coverage rate in 2021 | 1.564 (1.221, 1.906) | **<0.001** | 1.440 (1.086, 1.794) | **<0.001** |
|  | exclude theme3 | Number of posts | -0.003 (-0.009, 0.003) | 0.257 | 0.000 (-0.004, 0.005) | 0.903 |
|  |  | The proportion of posts from females (%) | -0.011 (-0.066, 0.044) | 0.683 | -0.028 (-0.064, 0.007) | 0.115 |
|  |  | Coverage rate in 2021 | 1.575 (1.223, 1.927) | **<0.001** | 1.414 (1.054, 1.774) | **<0.001** |
| ***Number of posts annotated as each theme*** | total | Number of posts annotated as theme2 | 0.016 (0.001, 0.032) | **0.036** | -0.007 (-0.018, 0.004) | 0.205 |
|  |  | Number of posts annotated as theme4 | -0.012 (-0.098, 0.073) | 0.767 | 0.019 (-0.042, 0.081) | 0.520 |
|  |  | Number of posts annotated as theme5 | 0.006 (-0.041, 0.052) | 0.805 | 0.042 (0.010, 0.073) | **0.013** |
|  |  | Number of posts annotated as theme6 | -0.022 (-0.035, -0.009) | **0.002** | -0.007 (-0.017, 0.003) | 0.159 |
|  |  | Coverage rate in 2021 | 1.668 (1.373, 1.964) | **<0.001** | 1.445 (1.125, 1.766) | **<0.001** |
|  | exclude theme3 and theme4 | Number of posts annotated as theme2 | 0.018 (0.003, 0.033) | **0.022** | -0.006 (-0.017, 0.005) | 0.268 |
|  |  | Number of posts annotated as theme5 | -0.001 (-0.047, 0.045) | 0.950 | 0.049 (0.017, 0.080) | **0.004** |
|  |  | Number of posts annotated as theme6 | -0.023 (-0.035, -0.011) | **<0.001** | -0.006 (-0.015, 0.004) | 0.228 |
|  |  | Coverage rate in 2021 | 1.669 (1.387, 1.951) | **<0.001** | 1.485 (1.173, 1.798) | **<0.001** |
|  | exclude theme3 | Number of posts annotated as theme1 | 0.000 (-0.044, 0.045) | 0.983 | 0.038 (0.008, 0.067) | **0.015** |
|  |  | Number of posts annotated as theme2 | 0.017 (0.002, 0.032) | **0.027** | -0.006 (-0.017, 0.005) | 0.254 |
|  |  | Number of posts annotated as theme4 | -0.009 (-0.091, 0.073) | 0.825 | 0.030 (-0.029, 0.088) | 0.308 |
|  |  | Number of posts annotated as theme6 | -0.022 (-0.035, -0.009) | **0.002** | -0.005 (-0.016, 0.005) | 0.294 |
|  |  | Coverage rate in 2021 | 1.678 (1.352, 2.003) | **<0.001** | 1.330 (0.986, 1.675) | **<0.001** |
| ***All factors*** | total | GDP per capita (in 10,000 CNY) | 0.307 (0.049, 0.565) | **0.022** | 0.094 (-0.069, 0.257) | 0.243 |
|  |  | Out-of-pocket share of health expenditure (%) | 0.014 (-0.085, 0.113) | 0.771 | 0.103 (0.037, 0.168) | **0.004** |
|  |  | Proportion of population with tertiary education (%) | -0.248 (-0.414, -0.082) | **0.005** | -0.038 (-0.145, 0.070) | 0.477 |
|  |  | Number of community health service centers (per 100,000 population) | 0.134 (-1.777, 2.405) | 0.886 | -0.661 (-1.895, 0.573) | 0.279 |
|  |  | The proportion of posts from females (%) | -0.010 (-0.075, 0.056) | 0.764 | -0.007 (-0.049, 0.034) | 0.714 |
|  |  | Number of posts annotated as theme1 | 0.033 (-0.007, 0.073) | 0.098 | 0.027 (0.003, 0.052) | **0.028** |
|  |  | Number of posts annotated as theme4 | -0.046 (-0.118, 0.027) | 0.209 | -0.001 (-0.049, 0.047) | 0.965 |
|  |  | Coverage rate in 2021 | 1.465 (1.138, 1.793) | **<0.001** | 1.348 (1.042, 1.653) | **<0.001** |
|  | exclude theme3 and theme4 | GDP per capita (in 10,000 CNY) | 0.275 (-0.004, 0.554) | 0.053 | 0.155 (-0.026, 0.335) | 0.090 |
|  |  | Out-of-pocket share of health expenditure (%) | 0.008 (-0.114, 0.130) | 0.894 | 0.131 (0.053, 0.208) | **0.002** |
|  |  | Proportion of population with tertiary education (%) | -0.219 (-0.404, -0.033) | **0.023** | -0.031 (-0.154, 0.093) | 0.611 |
|  |  | Number of community health service centers (per 100,000 population) | 0.127 (-1.870, 2.123) | 0.897 | -1.069 (-2.369, 0.231) | 0.102 |
|  |  | The proportion of posts from females (%) | -0.002 (-0.049, 0.044) | 0.921 | -0.002 (-0.032, 0.029) | 0.919 |
|  |  | Number of posts annotated as theme6 | -0.004 (-0.019, 0.011) | 0.620 | 0.005 (-0.006, 0.015) | 0.360 |
|  |  | Coverage rate in 2021 | 1.590 (1.237, 1.943) | **<0.001** | 1.402 (1.042, 1.762) | **<0.001** |
|  | exclude theme3 | GDP per capita (in 10,000 CNY) | 0.307 (0.048, 0.566) | **0.022** | 0.092 (-0.073, 0.257) | 0.259 |
|  |  | Out-of-pocket share of health expenditure (%) | 0.016 (-0.088, 0.119) | 0.759 | 0.101 (0.032, 0.171) | **0.006** |
|  |  | Proportion of population with tertiary education (%) | -0.255 (-0.419, -0.090) | **0.004** | -0.041 (-0.148, 0.067) | 0.441 |
|  |  | Number of community health service centers (per 100,000 population) | 0.152 (-1.760, 2.067) | 0.870 | -0.647 (-1.892, 0.599) | 0.293 |
|  |  | The proportion of posts from females (%) | 0.000 (-0.047, 0.048) | 0.983 | -0.005 (-0.035, 0.026) | 0.753 |
|  |  | Number of posts annotated as theme1 | 0.034 (-0.006, 0.074) | 0.093 | 0.027 (0.003, 0.052) | **0.030** |
|  |  | Number of posts annotated as theme4 | -0.045 (-0.117, 0.027) | 0.208 | 0.000 (-0.048, 0.048) | 0.995 |
|  |  | Coverage rate in 2021 | 1.486 (1.165, 1.808) | **<0.001** | 1.362 (1.058, 1.665) | **<0.001** |
| CI: confidence interval  ^a^ All post count variables were standardized by the provincial number of internet users.  ^b^ Influential user is defined as user with more than 10,000 followers. | | | | | | |

| **Table S4. The retention frequency of candidate factors.** | | | | | | | | | | | | | | | | | |
| --- | --- | --- | --- | --- | --- | --- | --- | --- | --- | --- | --- | --- | --- | --- | --- | --- | --- |
| **Models** | | **Number of factors** | GDP per capita (in 10,000 CNY) | Health expenditure per capita (in 1,000 CNY) | Out-of-pocket share of health expenditure (%) | Proportion of population with tertiary education (%) | Unemployment rate (%) | Number of community health service centers (per 100,000 population) | Number of posts | The proportion of posts from females | Number of posts from influential users | Number of posts annotated as theme1 | Number of posts annotated as theme2 | Number of posts annotated as theme3 | Number of posts annotated as theme4 | Number of posts annotated as theme5 | Number of posts annotated as theme6 |
| ***Female aged 9–45, one-dose (%)*** | | | | | | | | | | | | | | | | | |
| Socioeconomic factors | | 6 | 31 | 2 | 29 | 31 | 0 | 29 |  |  |  |  |  |  |  |  |  |
| All posts ^a^, posts from influential users ^b^ and the proportion from females | total | 3 |  |  |  |  |  |  | 31 | 31 | 0 |  |  |  |  |  |  |
|  | exclude theme3 and theme4 | 3 |  |  |  |  |  |  | 31 | 31 | 0 |  |  |  |  |  |  |
|  | exclude theme3 | 3 |  |  |  |  |  |  | 31 | 31 | 0 |  |  |  |  |  |  |
| Number of posts annotated as each theme | total | 6 |  |  |  |  |  |  |  |  |  | 1 | 30 | 1 | 31 | 30 | 31 |
|  | exclude theme3 and theme4 | 4 |  |  |  |  |  |  |  |  |  | 4 | 30 |  |  | 27 | 31 |
|  | exclude theme3 | 5 |  |  |  |  |  |  |  |  |  | 30 | 30 |  | 31 | 1 | 31 |
| All factors | total | 15 | 31 | 0 | 29 | 31 | 0 | 29 | 0 | 31 | 0 | 25 | 2 | 0 | 31 | 6 | 1 |
|  | exclude theme3 and theme4 | 13 | 31 | 1 | 29 | 29 | 0 | 29 | 0 | 31 | 2 | 15 | 3 |  |  | 15 | 28 |
|  | exclude theme3 | 14 | 31 | 0 | 29 | 31 | 0 | 29 | 0 | 31 | 0 | 30 | 2 |  | 31 | 1 | 1 |
| ***Female aged 9–45, three-dose (%)*** | | | | | | | | | | | | | | | | | |
| Socioeconomic factors | | 6 | 31 | 2 | 29 | 31 | 0 | 30 |  |  |  |  |  |  |  |  |  |
| All posts, posts from influential users and the proportion from females | total | 3 |  |  |  |  |  |  | 31 | 31 | 0 |  |  |  |  |  |  |
|  | exclude theme3 and theme4 | 3 |  |  |  |  |  |  | 31 | 31 | 0 |  |  |  |  |  |  |
|  | exclude theme3 | 3 |  |  |  |  |  |  | 31 | 31 | 0 |  |  |  |  |  |  |
| Number of posts annotated as each theme | total | 6 |  |  |  |  |  |  |  |  |  | 1 | 30 | 1 | 31 | 30 | 31 |
|  | exclude theme3 and theme4 | 4 |  |  |  |  |  |  |  |  |  | 4 | 30 |  |  | 27 | 31 |
|  | exclude theme3 | 5 |  |  |  |  |  |  |  |  |  | 30 | 30 |  | 31 | 1 | 31 |
| All factors | total | 15 | 31 | 0 | 29 | 31 | 0 | 30 | 0 | 31 | 0 | 25 | 2 | 0 | 31 | 6 | 1 |
|  | exclude theme3 and theme4 | 13 | 31 | 1 | 29 | 29 | 0 | 30 | 0 | 31 | 2 | 15 | 3 |  |  | 15 | 28 |
|  | exclude theme3 | 14 | 31 | 0 | 29 | 31 | 0 | 30 | 0 | 31 | 0 | 30 | 2 |  | 31 | 1 | 1 |

^a^ All post‑count variables were standardized by the provincial number of internet users.

^b^ Influential user is defined as user with more than 10,000 followers.

# **Figure S1. Flowchart of Weibo posts screening.**

| **Table S5. Sensitivity analysis (VIF cutoff of 5) for the model of HPV vaccination coverage for Weibo posts and socioeconomic factors in 31 provinces in China.** | | | | | |
| --- | --- | --- | --- | --- | --- |
| **Full models** | **Variable ^a^** | **Female aged 9–45,**  **one-dose (%)** | | **Female aged 9–45,**  **three-dose (%)** | |
|  |  | **Regression coefficient (95% CI)** | ***P*** | **Regression coefficient (95% CI)** | ***P*** |
| **Exclude theme3 and theme4** | GDP per capita (in 10,000 CNY) | 0.169 (-0.113, 0.450) | 0.229 | 0.126 (-0.032, 0.283) | 0.113 |
|  | Out-of-pocket share of health expenditure (%) | 0.059 (-0.057, 0.176) | 0.306 | 0.119 (0.049, 0.188) | **0.002** |
|  | Number of community health service centers (per 100,000 population) | -1.760 (-3.482, -0.038) | **0.045** | -1.051 (-2.075, -0.026) | **0.045** |
|  | The proportion of posts from females (%) | -0.005 (-0.056, 0.046) | 0.836 | -0.004 (-0.034, 0.025) | 0.755 |
|  | Coverage rate in 2021 | 1.374 (1.090, 1.659) | **<0.001** | 1.500 (1.260, 1.739) | **<0.001** |
| **Exclude theme3** | GDP per capita (in 10,000 CNY) | 0.156 (-0.138, 0.451) | 0.285 | 0.076 (-0.075, 0.227) | 0.312 |
|  | Out-of-pocket share of health expenditure (%) | 0.061 (-0.060, 0.181) | 0.310 | 0.106 (0.041, 0.172) | **0.003** |
|  | Number of community health service centers (per 100,000 population) | -1.771 (-3.527, -0.051) | **0.048** | -0.931 (-1.878, 0.015) | 0.053 |
|  | The proportion of posts from females (%) | -0.002 (-0.060, 0.055) | 0.938 | -0.005 (-0.035, 0.024) | 0.710 |
|  | Number of posts annotated as theme1 | 0.009 (-0.035, 0.053) | 0.673 | 0.025 (0.004, 0.047) | **0.024** |
|  | Coverage rate in 2021 | 1.335 (0.967, 1.702) | **<0.001** | 1.333 (1.070, 1.597) | **<0.001** |

HPV: human papillomavirus, CI: confidence interval

^a^ As presented in table3, the selection process resulted in variables from the initial candidate factors, and the coverage rate in 2021 was controlled.

^b^ All post count variables were standardized by the provincial number of internet users.

| **Table S6. Data on HPV vaccine coverage and socioeconomic characteristics across regions.** | | | | | | | | | | |
| --- | --- | --- | --- | --- | --- | --- | --- | --- | --- | --- |
| **REGION** | Total population ^a^ (100,000 persons) | Number of internet users ^b^ (10,000 persons) | One dose (%) | Three doses (%) | per capita ^a^ (CNY) | Health expenditure per capita ^c^ (CNY) | Out-of-pocket share of health expenditure ^c^ (%) | Number of individuals with junior college degree or above ^d^  (/100,000 persons) | unemployment rate ^e^(%) | Number of community health service centers ^f^ (/100,000 persons) |
| Beijing | 2184 | 1959 | 25.40 | 20.29 | 190313 | 16707 | 13.56 | 41980 | 3.2 | 1.607142857 |
| Tianjin | 1363 | 1145 | 13.67 | 9.50 | 119235 | 8145 | 26.14 | 26940 | 3.7 | 0.961115187 |
| Hebei | 7420 | 5978 | 8.95 | 5.13 | 56995 | 4861 | 28.63 | 12418 | 3.1 | 0.464959569 |
| Shanxi | 3481 | 2770 | 6.27 | 3.85 | 73675 | 4899 | 28.92 | 17358 | 2.3 | 0.669347889 |
| Inner Mongolia | 2401 | 1933 | 6.60 | 4.22 | 96474 | 6183 | 28.62 | 18688 | 3.8 | 1.441066222 |
| Liaoning | 4197 | 3488 | 7.12 | 4.28 | 68775 | 4900 | 28.59 | 18216 | 4.3 | 0.934000477 |
| Jilin | 2348 | 1845 | 7.99 | 5.24 | 55347 | 5272 | 27.93 | 16738 | 3.3 | 1.039182283 |
| Heilongjiang | 3099 | 2436 | 3.97 | 2.17 | 51096 | 5895 | 28.88 | 14793 | 3.2 | 1.526298806 |
| Shanghai | 2475 | 2224 | 20.99 | 14.49 | 179907 | 16177 | 13.91 | 33872 | 2.7 | 1.402020202 |
| Jiangsu | 8515 | 6426 | 13.05 | 8.44 | 144395 | 7691 | 23.43 | 18663 | 2.5 | 0.682325308 |
| Zhejiang | 6577 | 5030 | 14.30 | 9.76 | 118496 | 7747 | 22.53 | 16990 | 2.6 | 0.779990877 |
| Anhui | 6127 | 4737 | 8.65 | 5.33 | 73603 | 4592 | 28.50 | 13280 | 2.5 | 0.602252326 |
| Fujian | 4188 | 3208 | 13.75 | 6.56 | 126829 | 5401 | 24.81 | 14148 | 3.3 | 0.573065903 |
| Jiangxi | 4528 | 3200 | 4.99 | 3.16 | 70923 | 4443 | 27.63 | 11897 | 2.8 | 0.406360424 |
| Shandong | 10163 | 7714 | 11.70 | 7.40 | 86003 | 5710 | 27.35 | 14384 | 2.9 | 0.604152317 |
| Henan | 9872 | 7476 | 8.98 | 5.12 | 62106 | 4515 | 29.13 | 11744 | 3.4 | 0.582455429 |
| Hubei | 5844 | 4247 | 10.14 | 5.84 | 92059 | 5790 | 27.48 | 15502 | 3.0 | 0.626283368 |
| Hunan | 6604 | 4929 | 6.92 | 3.94 | 73598 | 5117 | 27.20 | 12239 | 2.3 | 0.643549364 |
| Guangdong | 12657 | 9709 | 15.65 | 8.63 | 101905 | 7022 | 24.87 | 15699 | 2.5 | 0.993126333 |
| Guangxi | 5047 | 3730 | 9.37 | 5.54 | 52164 | 4125 | 27.39 | 10806 | 2.5 | 0.39825639 |
| Hainan | 1027 | 716 | 14.04 | 7.15 | 66602 | 5705 | 20.45 | 13919 | 3.1 | 0.652385589 |
| Chongqing | 3213 | 2378 | 12.16 | 8.29 | 90663 | 5839 | 26.98 | 15412 | 2.9 | 0.778089013 |
| Sichuan | 8374 | 5958 | 11.36 | 7.61 | 67777 | 5601 | 26.91 | 13267 | 3.6 | 0.654406496 |
| Guizhou | 3856 | 2617 | 4.74 | 2.74 | 52321 | 4110 | 24.14 | 10952 | 4.5 | 0.848029046 |
| Yunnan | 4693 | 3135 | 6.31 | 3.66 | 61716 | 4679 | 26.94 | 11601 | 3.8 | 0.449605796 |
| Xizang | 364 | 252 | 3.51 | 0.88 | 58438 | 7596 | 8.52 | 11019 | 2.6 | 0.32967033 |
| Shaanxi | 3956 | 2941 | 11.67 | 7.12 | 82864 | 6088 | 28.46 | 18397 | 3.5 | 0.62942366 |
| Gansu | 2492 | 1612 | 3.41 | 2.16 | 44968 | 4522 | 27.92 | 14506 | 3.4 | 0.866773676 |
| Qinghai | 595 | 417 | 2.95 | 1.78 | 60724 | 6703 | 21.11 | 14880 | 1.8 | 0.588235294 |
| Ningxia | 728 | 520 | 7.03 | 4.50 | 69781 | 6230 | 24.85 | 17340 | 4.1 | 0.604395604 |
| Xinjiang | 2587 | 2015 | 2.55 | 1.27 | 68552 | 6664 | 21.86 | 16536 | 2.0 | 0.800154619 |

^a^ This data is for 2022 and sourced from the China Statistical Yearbook 2023. The access URL is provided below: https://www.stats.gov.cn/sj/ndsj/2023/indexch.htm

^b^ This data is for 2022 and sourced from the The 51st Statistical Report on Internet Development in China. The access URL is provided below:

https://cnnic.cn/NMediaFile/2023/0322/MAIN16794576367190GBA2HA1KQ.pdf

^c^ This data is for 2020 and sourced the 7th National Population Census of China. The access URL is provided below: https://www.stats.gov.cn/sj/ndsj/2024/indexch.htm

^d^ This data is for 2020 and sourced the 7th National Population Census of China. The access URL is provided below: https://www.stats.gov.cn/sj/pcsj/rkpc/7rp/indexch.htm

^e^ This data is for 2021 and sourced from the China Statistical Yearbook 2022. Official unemployment rate data have not been updated thereafter. The access URL is provided below: https://www.stats.gov.cn/sj/ndsj/2022/indexch.htm

^f^ This data is for 2022 and sourced from the China Statistical Yearbook 2022. Official unemployment rate data have not been updated thereafter. The access URL is provided below: https://www.nhc.gov.cn/mohwsbwstjxxzx/tjtjnj/202501/8193a8edda0f49df80eb5a8ef5e2547c

# **Table S7. A Custom Lexicon for Long Text Segmentation in Weibo Posts (in Chinese).**

| bivalent | quadrivalent | nonavalent | 9-valent | 2-valent |
| --- | --- | --- | --- | --- |
| 4-valent | valence | injection | 9 years old | 26 years old |
| 45 years old | hpv | HPV | vaccine | cervix |
| anal cancer | oropharyngeal cancer | genital warts warts | cancer | sexual transmission |
| free | vaccination | prevention | immunity | 9-valent HPV |
| hpv vaccine | HPV vaccine | age | subscribe | line up |
| wait | rush | expensive | price | beyond age limit |
| suggestion | proposal | implement | policy | extended age range |
| restriction | eligible age | infection | sequelae | side effects |
| research | science popularization | sales drive | scalper | desire |
| target | plan | opportunity | must-do | menstruation |
| sore | ache | pain | dizzy | swollen |
| bloated | sleepy | medical insurance | cost | carry out |
| take (shot) | sexual intercourse | bump | half a year | successfully vaccinated |
| experience | finally | health | anxiety | difficult |
| lottery | wish | gift | get selected |  |
